# Edaravone Plays Protective Effects on LPS-Induced Microglia by Switching M1/M2 Phenotypes and Regulating NLRP3 Inflammasome Activation

**DOI:** 10.3389/fphar.2021.691773

**Published:** 2021-05-31

**Authors:** Jiping Li, Xinping Dai, Liuyi Zhou, Xinxiu Li, Dongxiao Pan

**Affiliations:** ^1^ Department of Neurosurgery, HwaMei Hospital, University of Chinese Academy of Sciences, Ningbo, China; ^2^ Department of Emergency, Ningbo Yinzhou No.2 Hospital, Ningbo, China; ^3^ Operating Room, Ningbo Yinzhou No.2 Hospital, Ningbo, China; ^4^ Department of Experimental Medical Science, HwaMei Hospital, University of Chinese Academy of Sciences, Ningbo, China

**Keywords:** parkinson’s disease, microglia, inflammasome, edaravone, polarization

## Abstract

Parkinson’s disease is a neurodegenerative disorder in which activated microglia may appear prior to motor symptoms, but the specific therapeutic mechanisms remain unclear. This study investigated the potential effects of Edaravone (EDA) on M1/M2 polarization of microglia in rats with dopaminergic neurons damage induced by lipopolysaccharide (LPS) and its mechanism. Rats were randomly grouped as the following (*n* = 10): Control, EDA alone (10 mg/kg), LPS-model (LPS 5 μg), LPS + EDA (5 mg/kg) and LPS + EDA (10 mg/kg). After intragastric administration of EDA once a day for seven consecutive days, LPS was injected into SN pars unilaterally. Rotarod test, pole test, and traction test were used to analyze the intervention effect of EDA on neurobehavioral function in rats. Protein expression levels of TH, TNF-α, Arg-1, Iba-1, NLRP3 and caspase-1 were measured by immunofluorescence staining and western blot. *In vitro*, BV-2 cells were treated with LPS (100 ng/ml) before adding different doses of EDA. Levels of inflammatory cytokines in culture medium were detected by ELISA. Western blot and immunofluorescence were used to evaluate microglial activation and polarization. First, rotarod test, pole test, and traction test all showed that EDA mitigated motor dysfunction of PD rats. Second, pathological analysis suggested that EDA inhibited LPS-induced microglial activation and remitted declines of dopaminergic neurons. In addition, EDA shifted M1 pro-inflammatory phenotype of microglia to M2 anti-inflammatory state, while decreased expression of M1 markers (TNF-α and IL-1β) and facilitated expression of M2 markers (Arg-1 and IL-10). EDA suppressed inflammatory responses through inhibiting the expression of pro-inflammatory factors (IL-1β, IL-18 and NO), but the neuroprotective effects were invalid while siRNA NLRP3 existed. In conclusion, these results indicated that EDA could improve neurobehavioral functions and play anti-neuroinflammatory roles in PD rats, possibly by inhibiting NLPR3 inflammasome activation and regulating microglia M1/M2 polarization.

## Introduction

Parkinson’s disease (PD) is an age-related neurodegenerative disease around the world, whose pathogenesis is still unclear (Zou et al., 2015). Clinical signs include progressive dyskinesia with phenotypic severity, resting tremor, rigidity and bradykinesia, and may be accompanied with gastrointestinal dysfunction (Jankovic, 2008; Skjaerbaek, 2021). Classical pathological feature is the progressive loss of dopaminergic (DAergic) neurons within the nigrostriatal tract, related to decreased dopamine content in the striatum, which exacerbates motor function damage of patients. The prevalence of PD are unknown although the incidence is similar between Europeans and Han Chinese populations (Von Campenhausen et al., 2005; De Lau and Breteler, 2006). According to the clinical and etiological overlaps between PD and inflammation, recent preclinical researches have indicated that there are strong correlations between activated microglia and the progression of PD symptoms. Dramatically, postmortem studies in humans have shown the relationships between activated microglia and degeneration of dopaminergic neurons in nigra (Overmyer et al., 1999). However, once the stimuli occur in the brain, major innate immune cells in the brain will be activated and infiltrated locally to maintain homoeostasis of the lesion. Activated microglia are usually divided into two polarization states, termed “M1” (pro-inflammatory) phenotype and “M2” (anti-inflammatory) phenotype. In addition, in patients with neurodegenerative diseases such as Alzheimer’s disease and Parkinson’s disease, the proportion of M1-polarized microglia increased. For simplicity, activated M1-type (classical) and activated M2-type (alternative) microglia models have has been widely used *in vitro*. Generally, M1-phenotype microglia are considered to have pro-inflammatory activity by increasing production of inflammatory cytokines, such as interleukin-1β (IL-1β) and tumor necrosis factor-α (TNF-α), leading to tissue damage. On the contrary, M2-phenotype microglia exert a neuroprotective effect by upregulating anti-inflammatory mediators and suppressing the pro-inflammatory response. Activated microglia can coexist as single or mixed phenotype, which reflects the complexity of microglia function and the dynamic changes of the environment *in vivo* (Sica and Mantovani, 2012).

Increasing studies demonstrated that the release of neurotoxic factors of microglia might be associated with the NLRP3 Inflammasome (Gustin et al., 2015). The NOD-like receptor family, pyrin domain containing-3 protein (NLRP3) is a multi-protein complex that can regulate the maturation and secretion of the pro-inflammatory cytokines, including IL-1β and interleukin-18 (IL-18). Existing studies have demonstrated that inhibition of the NLRP3 Inflammasome activation could protect dopaminergic neurons (Gao et al., 2002; Gustin et al., 2015; Sarkar et al., 2017; Abas et al., 2018). Based on this theory, researchers are extensively seeking for new compounds to inhibit the activation of NLRP3 Inflammasome for therapeutic intervention against neurodegeneration in PD. Epidemiological studies have shown that chronic patients who use anti-inflammatory drugs have a low risk of developing idiopathic PD (Honglei Chen et al., 2003; Esposito et al., 2007). Since the neuroinflammation play an important role in the pathogenesis of PD, anti-inflammatory drugs are considered as candidates for the treatment of PD (Faust et al., 2009; Wang et al., 2017a).

**FIGURE 1:**
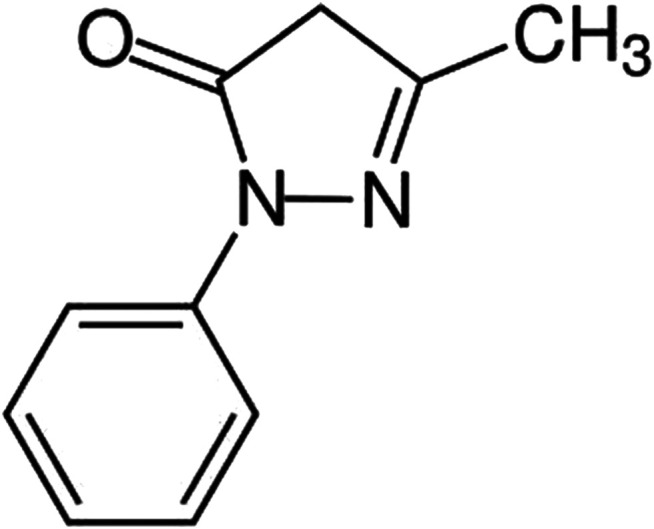
Chemical structures of edaravone (EDA).

Edaravone (3-methyl-1-phenyl-2-pyrazolin-5-one, EDA, Figure 1) is an ROS scavenger with a variety of biological activities such as anti-oxidative Suehiro et al. (2018), anti-inflammatory Zhang et al. (2019) and anti-tumor (Suehiro et al., 2018). Previous studies suggested that EDA could protect normal human bronchial epithelium from 6-OHDA-induced injury and improve motor function of rats through its anti-inflammation effect (Yuan et al., 2008). However, whether EDA can protect LPS-induced PD through mediating NLRP3 Inflammasome remains to be investigated. Based on the anti-inflammatory effect of EDA, we scrutinized whether EDA could inhibit microglia activation and alleviate dopaminergic neurodegeneration in PD models *in vivo* and *in vitro*. Previous study had showed that EDA could attenuate amyloid-β-induced proinflammatory response by inhibiting NLRP3 Inflammasome (Moehle and West, 2014). EDA has also been reported to have a therapeutic effect on retinal injury through regulating nuclear factor κB (NF-κB) signaling pathways in diabetic rats (Wan et al., 2019). Interestingly, EDA also could cross the blood–brain barrier (BBB) (Fang et al., 2014) and maintain integrity of BBB by activating the NFE2-related factor 2 signaling pathway (Liu et al., 2019). However, there is currently no such evidence for the role of EDA between microglia and dopaminergic neurons in inflammation.

The current study aims to identify the role of EDA in microglial polarization in PD, and to provide a basis for microglial-based diagnostic and therapeutic strategies. Specifically, these findings might give an insight into a potential therapeutic target for treating PD.

## Materials and Methods

### Reagent

Edaravone (≥ 99%) and lipopolysaccharide were purchased from Sigma-Aldrich (St. Louis, MO, United States). Primary antibodies against tyrosine hydroxylase, Iba-1, NLRP3, caspase-1, ASC, β-actin, Rabbit IgG and mouse IgG antibodies were bought from Proteintech Group (Chicago, IL, United States). Primary antibodies anti-Arg-1, anti-TNF-αand anti-CD11b/c were supplied by Abcam (Cambridge, MA, United States). MTT assay kit was obtained from Beijing Solarbio science and Technology Co., Ltd. (Beijing, China). Enzyme-linked Immunosorbant Assay (ELISA) for TNF-α, IL-1β and IL-18 were bought from Elabscience Biotechnology Co., Ltd. (Wuhan, China). Dulbecco’s modified eagle medium (DMEM) and OPTI-MEM were provided by Gibco-BRL (Invitrogen Life Technologies, Carlsbad, United States). The small interfering RNA (siRNA) against NLRP3 was purchased from GenePharma (Shanghai, China).

### Animal and Treatment

A total of 50 adult male Sprague-Dawley rats (200–230 g) were purchased from the Experimental Animal Center of the Nanjing Medical University (Nanjing, China; Specific pathogen-free Grade II; Certificate No. SCXK 2018-0011). All the animals were housed in a temperature (25 ± 2°C), light (12 h of daylight, 12 h of darkness) and humidity (60 ± 4%) environment. Adequate food and water were provided and available at any time. Rats were allowed to adapt to the environment for 1 week before experiments.

All the animals were randomly allocated to five experimental groups (*n* = 10): Control (0.9% NaCl), EDA alone (10 mg/kg), LPS-model (LPS 5 μg), LPS + EDA (5 mg/kg) and LPS + EDA (10 mg/kg). DA neuronal damage was induced by receiving unilateral injection of LPS (5 μg) into the SN pars compacts, and the coordinate position as described in previous studies (Fang et al., 2014). After daily intragastric administration of EDA for 7 days, behaviors of rats were analyzed by rotarod test, pole test, and traction test. Then all animals devoted their lives and tissues were collected for subsequent experiments.

### Cell Culture and Treatment

BV-2 cells, a murine microglia cell line, were purchased from Wuhan University Cell Library (Wuhan, China). BV-2 cells were cultured in DMEM medium with 10% fetal bovine serum (FBS) and 1% penicillin-streptomycin in a incubator with 5% CO2 at 37°C. BV-2 cells were pretreated with different doses of EDA, and 1 h later, LPS was added to the medium for 24 h. Then, the culture medium and protein were used for later experiments.

### MTT Assay

BV-2 cells were seeded into 96-well plates at a density of 2 × 10^4^/ml for 24 h, then the supernatant was replaced by new medium containing 2% FBS, and the cells were exposed to LPS with or without EDA for another 24 h. After that, 3-(4,5-dimethylthiazol-2-yl)-2,5-diphenyltetrazoliumbromide (MTT) was added for 4 h to verify cell viability. Using the microplate reader to measure the optical density values at 490 nm.

### Enzyme-Linked Immunosorbent Assay

According to the manufacturer’s instructions, the levels of TNF-α, IL-1β and IL-18 were measured by ELISA (R&D Systems. United States). The microplate reader (Sunnyvale, CA, United States) was used to assay the absorbance at 450 nm, And the experiment was repeated at least three times.

### Western Blot

The total protein content was extracted from midbrain tissue and BV2 cells by using a lysis buffer containing protease inhibitors after washing with PBS three times. The protein concentration was measured with the BCA protein assay kit (Solarbio, pc0020). Equal amounts of protein were separated by sodium dodecyl sulfate/polyacrylamide gel electrophoresis and transferred to polyvinylidene fluoride (PVDF) membrane. The membrane was blocked in TBS (Solarbio) with Tween 20 (Sigma, P9416) and 5% skim milk for 2 h. Then, the PVDF was incubated with the following primary antibodies overnight at 4°C: TH (1:1,000), IBA-1 (1:800), NLRP3 (1:1,000), ASC (1:400), caspase-1 (1:800), Arg-1 (1:1,000), β-actin (1:2,000). After washing with TBST, the membrane was incubated with horseradish-peroxidase-conjugated anti-mouse IgG antibody or anti-rabbit IgG (1:2,000) for 1 h at room temperature. At last, the PVDF was treated with enhanced ECL reagent and protein bands were visualized on X-ray film utilizing enhance chemiluminescence system.

### Immunofluorescence

Culture medium was removed and the slices were treated with 0.3% Triton X-100 for 30 min to fix. Then, the cells/tissues were orderly blocked with goat serum for 2 h, incubated with primary antibodies overnight at 4°C: Iba-1 (1:1,000), OX-42 (1:800), NLRP3 (1:800), and incubated with fluorescent-conjugated secondary antibody for 1 h. An Olympus microscope (Olympusr, Tokyo, Japan) was used to capture the Digital images of SN TH-positive neurons.

### Real-Time RT-PCR Assay

Total RNA was extracted with trizol agent and purified with RNeasy kit. Iba-1, TNF-α, IL-1β, Arg-1, IL-10, and β-actin genes were amplified by using the forward and reverse primers. Real-time PCR was performed using a SYBR Green Supermix according to the instruction and then determined on a CFX96 real-time PCR detection system (Bio-Rad, CA, United States). The data analysis software provided with the system was used to normalize the target gene expression levels comparing with β-actin expression.

### RNA Transfection

BV-2 cells were chosen as an experimental model and transfected with siRNA oligos against NLRP3 (GenePharma, China). Cells were seeded in 24-well plates for 24 h. The mixture of RNA oligo and GP-siRNA-Mate Plus was used for siRNAs transfection. After 6 h of treatment, DMEM medium was instead of the transfection solution for an additional 24 h before subsequent test.

### Rotarod Test

Motor performance of rats was assessed by using a rotarod apparatus (RotaRod system). First, the rats were trained to adapt at a speed of 10 rpm/min before the experiment. After intragastric administration of EDA for 7 days, record the time of each rat spent on the rotary, with speed accelerating from 10 to 30 rpm in 5 min. Remove the rats which remain on the apparatus after 5 min and score their time as 5 min. Calculate rotation time for data analysis.

### Pole Test

To measure the bradykinesia for mice induced by LPS, a wooden pole (50 cm in length) with a ball of 1.25 cm radius placed on top was used in the pole test to determine climbing time for mice. Briefly, mice were placed on the top of a pole with a rough surface, and the different time taken by each mouse to get down completely was recorded. Results were repeated when mice stopped or climbed to the reverse direction. Each mouse was re-tested for three times one day and recorded as previously described.

### Traction Test

To evaluate muscle strength and equilibrium, A horizontal rope with a distance of 30 cm was used as described previously for determining the suspension time for mice. The time hanging on the wire was recorded and scored as the following criteria: 0–4 s recorded as 0 point; 5–9 s recorded as 1 point; 10–14 s recorded as 2 point; 15–19 s recorded as 3 point; 20–24 s recorded as 4 point; 25–29 s recorded as 5 point, and above 30 s recorded as 6 point. The average of the score was calculated after performing daily in triplicate.

### Statistics

Unless otherwise stated, all the data values represent means ± SEM. One-way analysis of variance was used to evaluate the significant differences between groups, and statistical analysis was performed by using the software Graphpad Prism (version 5.01). A value of *p < 0.05* was viewed as statistically significant.

## Results

### Edaravone Improved Lipopolysaccharide-Induced DA Neuron Lesion and Neurobehavioral Functions

**FIGURE 2:**
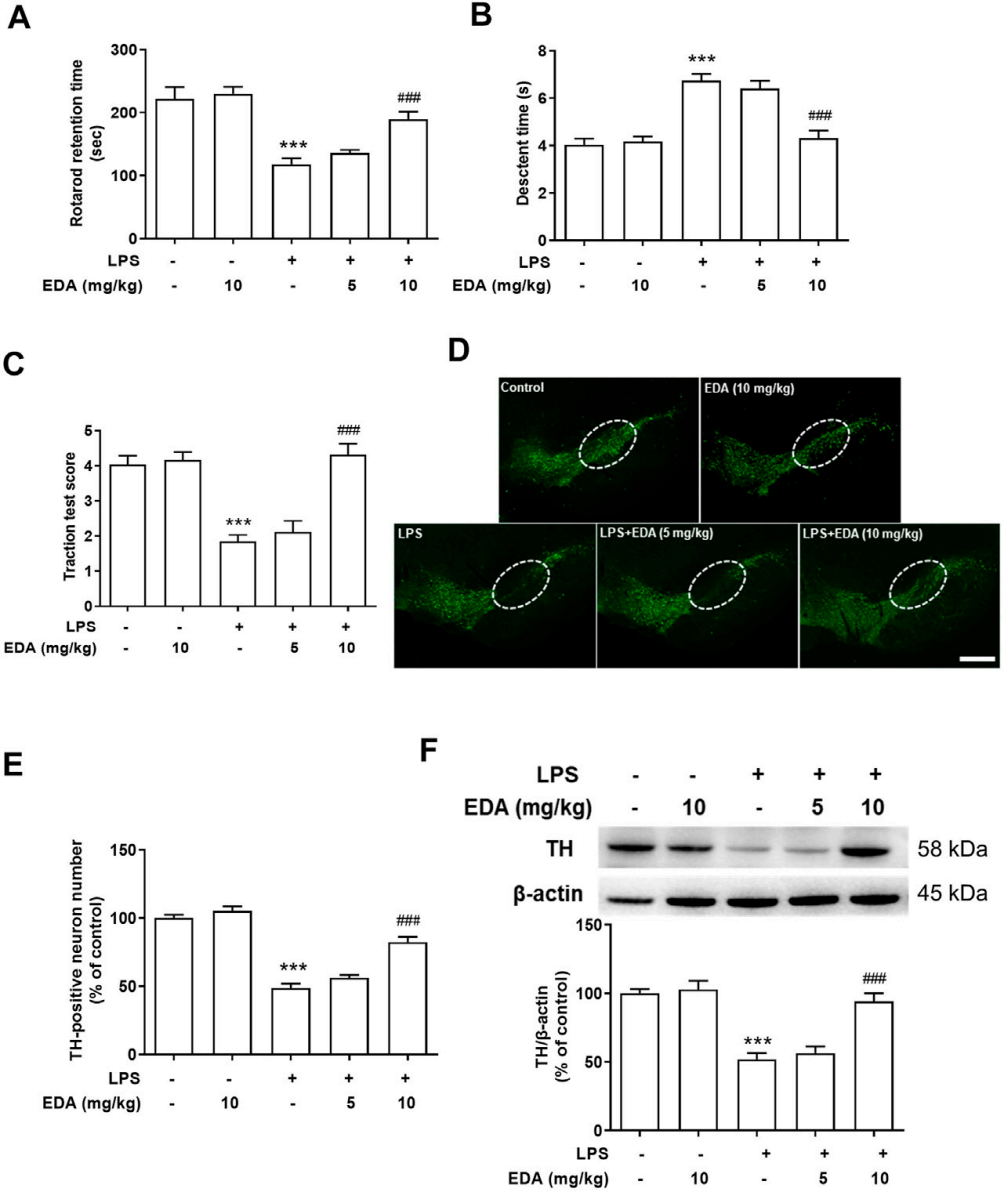
EDA attenuated LPS-induced DA neuron lesion and neurobehavioral functions *in vivo*. **(A)** Rotarod test: the time rats stayed on the rod was recorded. **(B)** Pole descent test: decline time was regarded as a measure of bradykinesia. **(C)** Traction test: traction reflex score was used to evaluate muscle strength and balance. **(D)** DA neuronal lesion was analyzed *via* the representative immunofluorescence staining for TH (green) in SN. The “ellipse” presented the area of SN. Scale bar = 200 μm. **(E)** The number of TH-positive neurons in SN was counted. **(F)** TH protein expression was detected by western blot assay. Data were expressed as a percentage of the control group and were the mean ± SEM from six rats. ***p < 0.001 compared with control group; ^###^ *p* < 0.001 compared with LPS group.

In order to verify the degree of motor dysfunction in LPS-induced DA neuron damage, a significant clinical symptoms of PD patients, the behavior of rats was analyzed by rotarod test, pole test and traction test after intragastric administration of EDA for 7 days. As shown in Figures 2A–C, LPS-induced PD model rats showed an obvious increase in locomotor deficiency compared with control rats in behavioral tests. At the same time, EDA significantly upregulated the time spent on the rod of LPS-induced rats. TH, a marker of TH^+^ dopaminergic neurons, acts as a rate-limiting enzyme in DA synthesis. Dopaminergic neurons survival in the SN were characterized by densitometric analysis. As shown in Figures 2D,E, there was no significant difference in the activity of DA neurons between the control group and EDA alone group, suggesting that EDA alone treatment had no effect on DA neurons. EDA ameliorated LPS-induced DA neuron loss in a dose-dependent manner, compared with LPS group. To further validate the neuroprotection effect of EDA on DA, the LPS-administrated protein expression of nigra TH was identified. As shown in Figure 2F, LPS reduced TH protein expression compared with control group and this reduction was weakened by EDA treatment.

### Edaravone Switched Microglial M1 to M2 Polarization *In Vivo*

**FIGURE 3:**
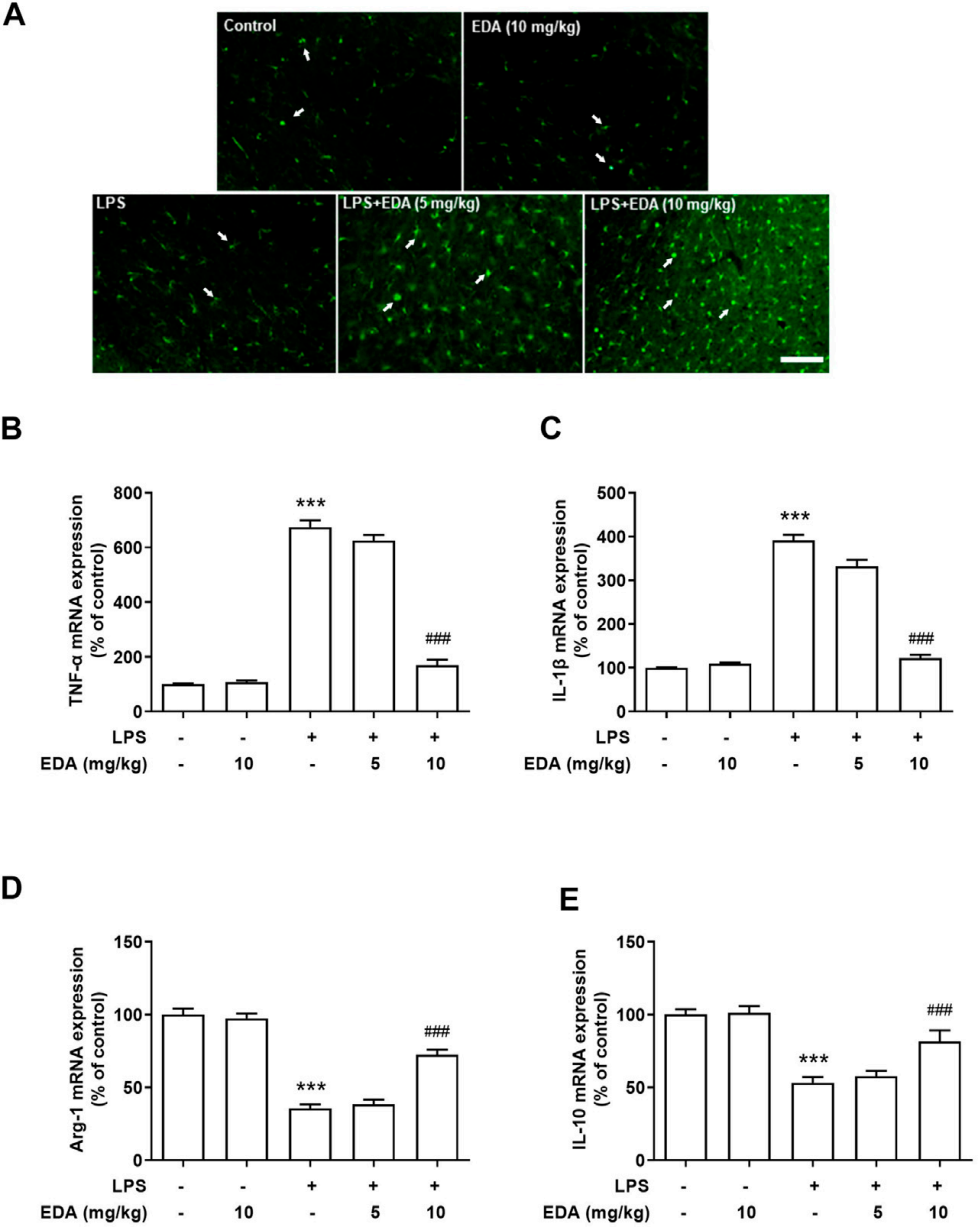
EDA switched microglial M1 to M2 polarization *in vivo*. **(A)**The expression of Arg-1 was detected in activated microglia in SN. **(B–C)** The mRNA expression of M1 markers TNF-α and IL-1β were detected after EDA treatment. **(D–E)** The mRNA expression of M2 markers Arg-1 and IL-10 were detected. Data were expressed as a percentage of the control group and were the mean ± SEM from six rats. ***p < 0.001 compared with control group; ^###^ *p* < 0.001 compared with LPS group.

To observe the effect of EDA on polarization of LPS-induced microglia, immunofluorescence analysis was used to assay the expression of Argnise-1 (Arg-1). As shown in Figure 3A, the protein expression of Arg-1 was located in activated microglia and EDA could enhanced LPS-induced microglial activation. Notably, strong TNF-α and less Arg-1 mRNA expressed were detected in LPS-treated rats. After EDA treatment, the mRNA expression of M1 markers TNF-α and IL-1β decreased, while the expressions of M2 markers of anti-inflammatory cytokines Arg-1 and IL-10 were enhanced shown in Figures 3B–E.

### EDA Attenuated LPS-Induced Microglial Activation *In Vitro*

**FIGURE 4:**
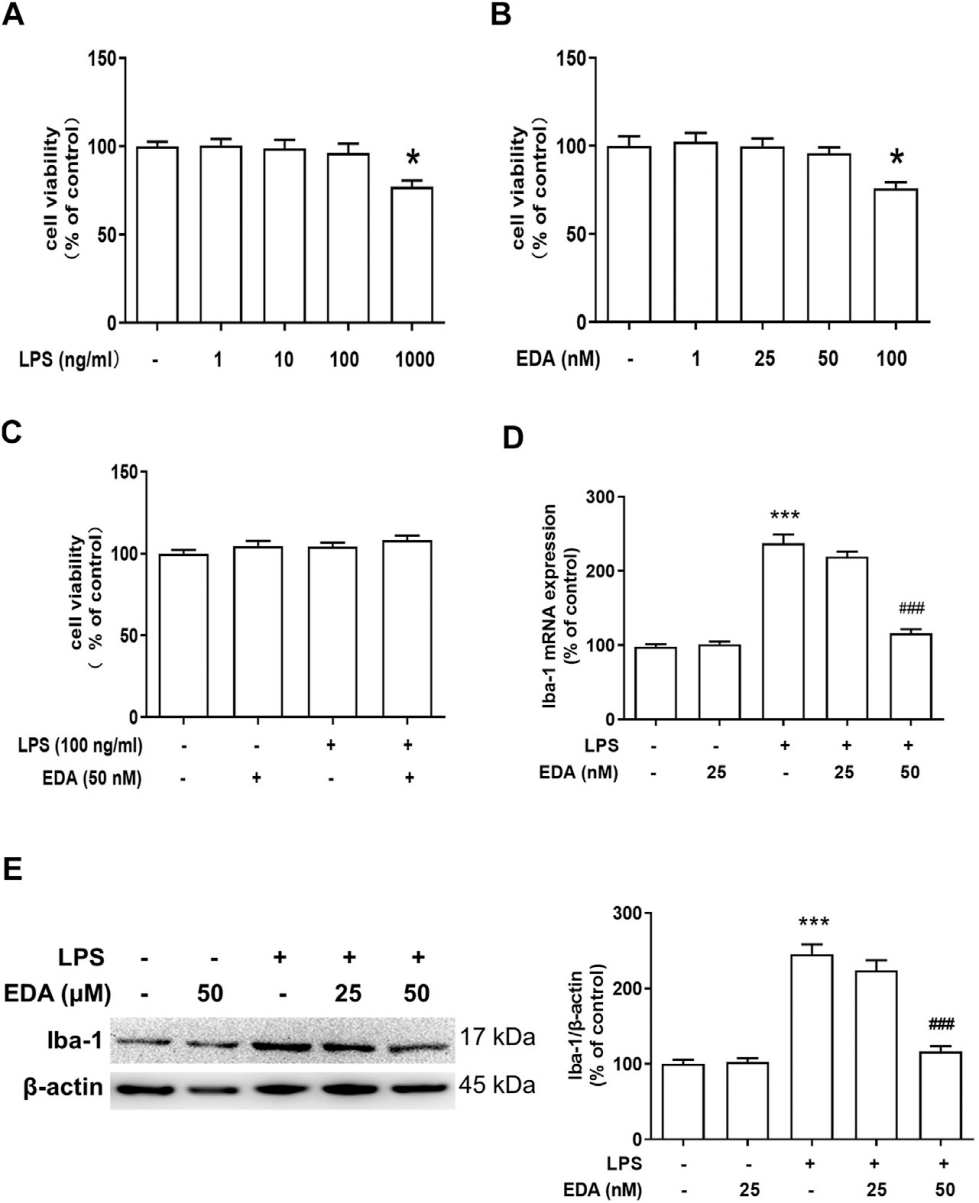
EDA attenuated LPS-induced microglial activation *in vitro*. **(A–C)** BV-2 cells were treated with different concentration of EDA and LPS for 24 h. Cell viability was determined by MTT assay. **(D)** The level of Iba-1 mRNA expression in BV-2 cell was measured *via* RT-PCR Assay. **(E)** Microglia activation was detected by western blot with an anti-Iba-1 antibody. Data were expressed as a percentage of the control group and were the mean ± SEM from six rats. *p < 0.05, ***p < 0.001 compared with control group; ^###^ *p* < 0.001 compared with LPS group.

To further identify the regulatory mechanism of EDA on activation of microglia, BV-2 cell line was employed to investigate the improvement effect of EDA on LPS-induced microglial activation *in vitro*. BV-2 cell was treated with different dose of EDA or/and LPS. First, MTT assay was applied to confirm the optimal concentration of EDA and LPS for subsequent experiments (Figures 4A–C), and the results indicated that the concentration of EDA (50 mM) and LPS (100 ng/ml) had no significant effects on cytotoxicity and viability of normal BV-2 cells. Secondly, to discern the function of EDA on microglia morphology, protein Iba-1 (a specific microglial marker, including resting microglia and activated microglia) was evaluated. As indicated in Figures 4D,E, western blot analysis and RT-PCR assay revealed that EDA suppressed LPS-induced Iba-1 protein and Iba-1 mRNA expression.

### Edaravone Switched Microglial M1 to M2 Polarization *In Vitro*

**FIGURE 5:**
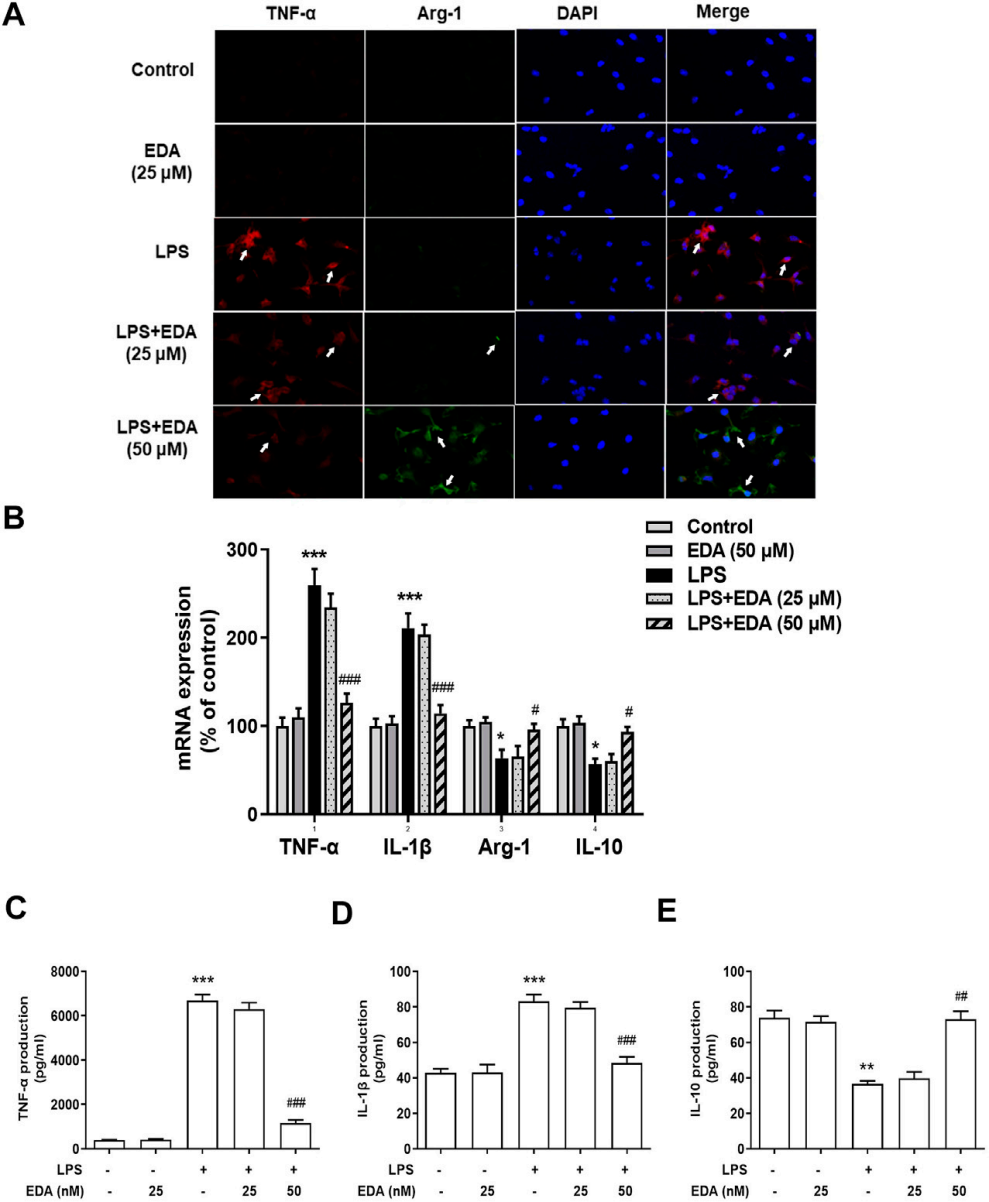
EDA switched Microglial M1 to M2 Polarization *in vitro*. BV-2 cells were pretreated with EDA for 60 min followed by LPS (100 ng/ml) adminstration for 24 h. **(A)** Cultures were visualized by immunostaining with anti-TNF-α and Arg-1 antibodies. **(B–E)** The levels of TNF-α, IL-1β and IL-10 in supernatant were detected by ELISA and the whole cells were collected to detect the gene expressions of TNF-α, IL-1β, Arg-1, and IL-10 by RT-PCR. Results were the mean ± SEM from six independent experiments performed in triplicate. *p < 0.05, ***p < 0.001 compared to the control cultures. ^#^ *p* < 0.05, ^###^ *p* < 0.001 compared to LPS-treated cultures. Scale bar = 50 mm

To explore the effect of EDA on LPS-stimulated phenotypic transformation of microglia, the expression of Arg-1 and TNF-α was detected by ELISA and RT-PCR (Figures 5A,B). The results showed that immunoreactivity of M1 marker (TNF-α) was in a high level and that of M2 marker (Arg-1) was low in LPS-treated cultures. However, TNF-α and Arg-1 expressions were significantly reversed in the EDA groups. This result suggested that EDA shifted the inflammatory M1 phenotype to the anti-inflammatory M2 phenotype.

The production of TNF-α, IL-10 and IL-1β in the cell supernatant were measured by ELISA, to assess the inhibitory actions of EDA on inflammatory cytokines expression and polarization of LPS-induced microglia. Then, the regulatory abilities of EDA on the production of M1 pro-inflammatory cytokines (TNF-α and IL-1β) and M2 anti-inflammatory cytokine were measured. As shown in Figures 5C–E, EDA reduced LPS-induced production of TNF-α, IL-1β in medium, while increased the production of IL-10 simultaneously.

### Edaravone Inhibited NLRP3 Inflammasome Activation

**FIGURE 6:**
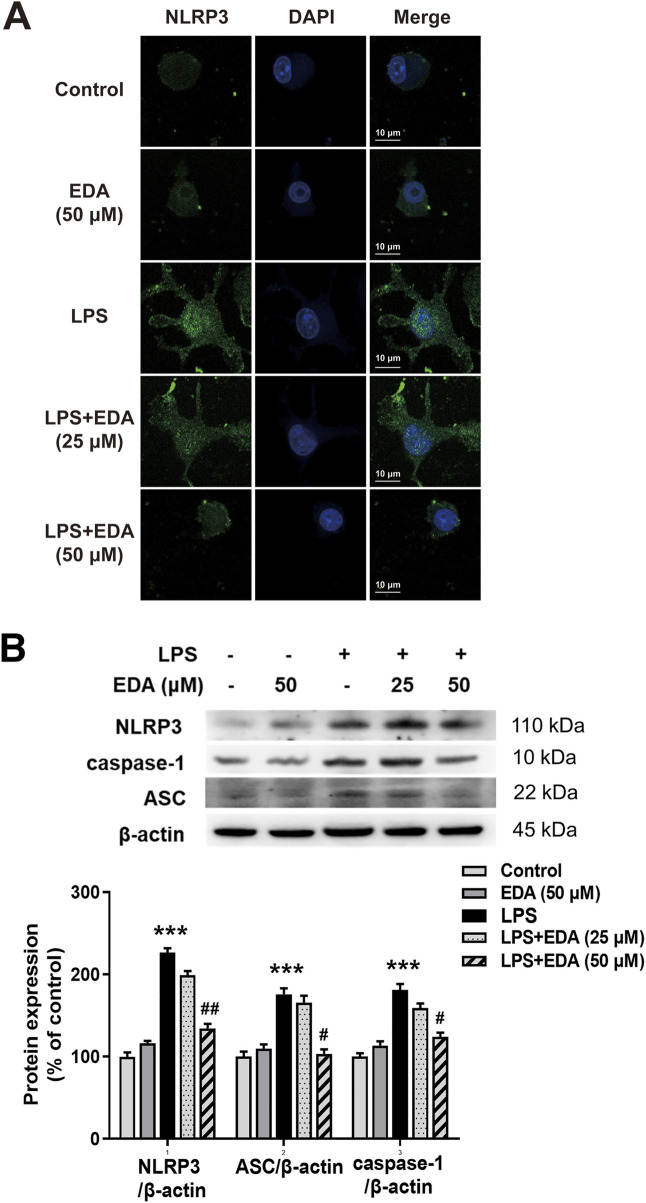
EDA Inhibited NLRP3 inflammasome activation *in vitro*. BV-2 cells were treated with EDA for 1 h followed by the application of LPS (100 ng/ml) for 24 h. **(A)** Immunofluorescence detection of NLRP3 inflammasome in BV-2 cells were performed. Scale bar = 10 μm. **(B)** The protein expressions of NLRP3, ASC and caspase-1 were detected by western blot assay. The ratio of densitometry values of NLRP3, ASC and caspase-1 with β-actin were assessed and normalized to each respective control cultures. Data were the mean ± SEM from six independent experiments performed in triplicate. ***p < 0.001 compared with control cultures; ^#^ *p* < 0.05, ^#^ *p* < 0.01 compared with LPS-treated cultures.

In order to investigate whether NLRP3 inflammasome was involved in the modulation of EDA in neuroinflammatory responses, the protein levels of NLRP3 were measured by immunofluorescence. As shown in Figure 6A, LPS significantly induced NLRP3 activation. Similarly, EDA inhibited LPS-induced increased expression of NLRP3, caspase-1, ASC proteins (Figure 6B).

### NLRP3 Inflammasome Participated in Edaravone-Mediated Microglial M1/M2 Polarization

**FIGURE 7:**
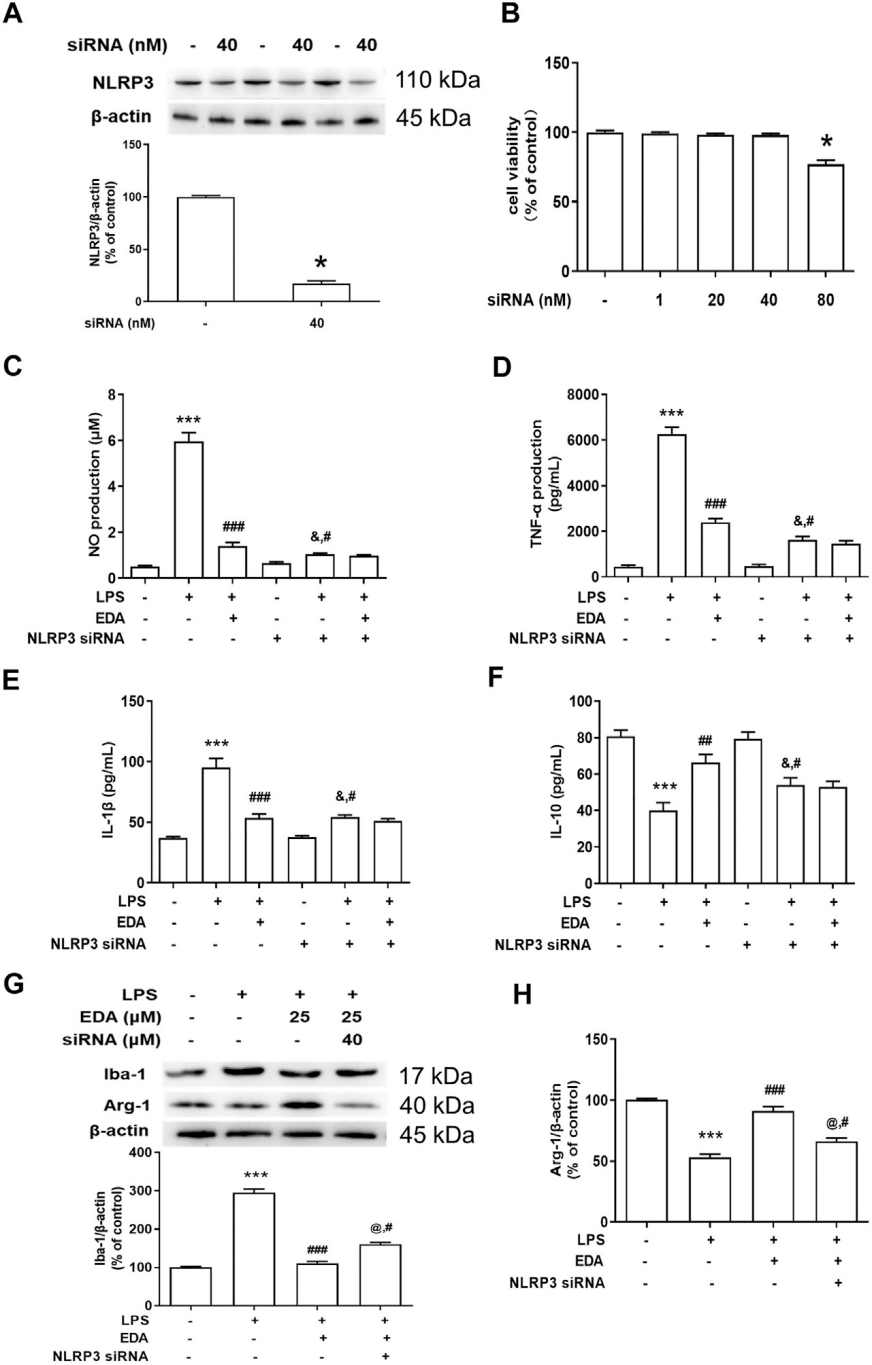
NLRP3 inflammasome participated in EDA-mediated microglial M1/M2 polarization. BV-2 cells were treated with NLRP3 siRNA (40 nmol/L). After 6 h of transfection, the transfection solution was removed and cells were rinsed with PBS. **(A)** The NLRP3 protein expression silence efficiency was validated *via* western blot assay in BV-2 cells. Then, cells were treated with EDA (50 μM) for 60 min and stimulated by LPS (1 μg/ml) for 24 h. **(B)** Cell viability was determined by MTT assay. **(C–F)** The production of IL-18, IL-1β and IL-10 in the culture medium was detected by ELISA, respectively. **(G–H)** The protein expression of Iba-1 and Arg-1 in BV-2 cells by western blot assay. The ratio of densitometry values of Iba-1 and Arg-1 was assessed and normalized to each respective control group. Data were the mean ± SEM from six independent experiments performed in triplicate. *p < 0.05, **p < 0.01, ***p < 0.001 compared with control cultures; ^#^ *p* < 0.05, ^##^ *p* < 0.01, ^###^ *p* < 0.001 compared with LPS-treated cultures. ^&^ *p* < 0.05 compared with NLRP3 siRNA alone-treated cultures.

To observe the link between NLRP3 inflammasome and EDA-mediated microglial M1/M2 polarization, NLRP3 siRNA (Nlrp3-Mus-727 siRNA oligo), a selective inhibitor of NLRP3 gene silencing, was applied in subsequent experiments. The successful silencing of NLRP3 expression with siRNA was certified by protein detection in BV2 cells (Figure 7A), and MTT assay indicated that NLRP3 siRNA (40 nM) didn’t affect cell viability (Figure 7B). In addition, NLRP3 siRNA significantly reduced the production of pro-inflammatory factors compared with LPS-treated cultures (Figures 7C–F).

Next, the role of NLRP3 inflammasome on M1/M2 polarization of microglia was conducted. As indicated in Figures 7G,H, compared to EDA-treated groups, results presented that the regulation of EDA on protein expression of Iba-1 and arg-1 was attenuated by NLRP3 siRNA.

## Discussion

This study showed that EDA could transform the microglia phenotype from pro-inflammatory M1 type to anti-inflammatory M2 type, thereby inhibit the microglia-mediated neuroinflammatory response. In addition, the effect of EDA on polarization state of microglia depends on the regulation of NLRP3 inflammasome. EDA can inhibit the M1-type polarization of microglia caused by NLRP3 activation, and further inhibit the inflammatory response involved by microglia. In summary, this study suggested that EDA regulated the M1/M2 polarization of microglia, and thus played an anti-neuroinflammatory role through inhibiting the activity of NLRP3 inflammasome (Graphical Abstract).

At present, the etiology of Parkinson's disease is thought to be related to oxidative stress, mitochondrial dysfunction, chronic neuroinflammation and other factors (Poewe et al., 2017; Adams et al., 2019). But the underlying mechanisms remain unclear. Quantity evidences indicate that neuroinflammation is associated with pathogenesis of neurological diseases. It has been reported that in neuroinflammation-mediated neurodegenerative diseases, nucleotide binding and oligomerization domain-like receptor pyrin domain contain three activated inflammatory bodies and microglia as well as released inflammatory cytokines such as IL-1β play important roles (Codolo et al., 2013). Inflammasome is an important part of the central immune system, which has three components: pattern recognition receptor (PRR), apoptosis-associated speck-like protein (ASC), and caspase-1 (Shuo et al., 2018). NLRP3 is an inflammatory complex, which exists in microglia, and its over-activation may lead to many diseases (Urwanisch et al., 2021). In the presence of stimuli, sustained activation of microglia will lead to the production of IL-1β and IL-18 mediated by NLRP3, both are powerful pro-inflammatory factors (Ramesh et al., 2013). Activated NLRP3 mediates autoproteolysis of caspase-1, then cleaves pro-IL-1β, whose evolution can activate IL-1β into an active state (Panicker et al., 2019). Studies have confirmed that mice without NLRP3 or caspase-1 are resistant to DA neuron loss in substantia nigra caused by rotenone and neurotoxin [1-methyl-4-phenyl-1,2,3,6-tetrahydropyradine (MPTP)] (Yan et al., 2015; Qiao et al., 2017). Studies have shown that activated microglia and NLRP3-rich inflammasomes have increased significantly in the substantia nigra of PD patients (Adams et al., 2019). Once being activated, microglia release pro-inflammatory and cytotoxic factors. The accumulation of toxic factors can cause damage to peripheral neurons. The dying neurons induce the secondary activation of microglia, which further damage the neurons. Recent studies also suggested natural compound, such as Silibinin and Astaxanthin, have beneficial effects in neuropathic pain by regulating oxidative-nitrosative stress and astrocyte activation (Fernandes et al., 2018; Sharma et al., 2018). Therefore, inhibiting the activation of microglia and inflammasome NLRP3 is a promising therapeutic strategy. EDA is a kind of active oxygen scavenger. Studies have shown that EDA can inhibit the production of active oxygen and microglia inflammation induced by Aβ (Wang et al., 2017b). This study shows that EDA can inhibit the neuroinflammatory response mediated by microglia activation. These results were consistent with previous studies that EDA relieved neuralgia by inhibiting neuroinflammation caused by microglia.

Microglia have high plasticity and can produce two morphologies of pro-inflammatory M1 phenotype and anti-inflammatory M2 phenotype under different stimuli. When microglia are stimulated by infection, trauma and other harmful stimuli, they will activate them into M1 phenotype and release TNF-α, IL-6 and IL-12, among which TNF-α is one of the markers of M1 phenotype microglia. This change result in the great enhancement of activated microglia phagocytosis. So, the activation of M1 phenotype microglia is a protective stress response of the body, but excessive activation can damage neurons. In contrast, the M2 phenotype is more friendly to nerve cells. After stimulating by IL-4, IL-10 etc., the microglia are polarized into the M2 phenotype, and express Arg1, which is the marker of the M2 phenotype microglia. M2 cells secrete anti-inflammatory factors that can reduce M1 activation and promote the body’s self-repair (Moehle and West, 2014). Based on the two opposing roles of microglial, it has long been debated whether the balance among the cytokines influence the final outcome from protective to destructive (Tiwari et al., 2014). In Parkinson’s disease, the M1 phenotype of microglia is over-expressed and the inflammatory response is over-enhanced, but inhibiting M1 activation alone does not provide protection (Dorsey et al., 2007). Promoting the polarization of microglia to the M2 phenotype and maintaining the balance of M1/M2 should be of great significance for the treatment of Parkinson’s disease. Due to the existence of BBB, commonly used anti-inflammatory drugs are often unsatisfactory in the treatment of neurological diseases. Most of compounds discovered so far can only improve inflammation by inhibiting M1 activation, and only a few compounds can promote the polarization of microglia to the M2 phenotype. This study showed that EDA can effectively inhibit the expression of microglia M1 phenotype markers and promote the M2 polarization of microglia, the anti-inflammatory phenotype, which may contribute to the neuroprotection. Moreover, EDA can cross the BBB, which creates favorable conditions for EDA to prevent and treat central nervous system diseases.

In summary, this study showed that EDA inhibited the activation of NLRP3 inflammasomes, reduced the M1 phenotype of microglia and promoted the cells to polarize to the M2 state, all these functions result in an improvement in neuroinflammatory response. The current research results support the potential drug application of EDA in the treatment of neuroinflammation-related neurological diseases.

## Conclusion

This study illustrated that EDA promoted microglial polarization towarding to M2 phenotype through inactivation of NLRP3 inflammasome. These findings provide a new evidence that EDA might have considerable value as a potent agent for neuroinflammatory diseases treatment.

## Data Availability

The raw data supporting the conclusions of this article will be made available by the authors, without undue reservation.
