# Correction: Edaravone plays protective effects on LPS-induced microglia by switching M1/M2 phenotypes and regulating NLRP3 inflammasome activation

**DOI:** 10.3389/fphar.2026.1774570

**Published:** 2026-04-22

**Authors:** Jiping Li, Xinping Dai, Liuyi Zhou, Xinxiu Li, Dongxiao Pan

**Affiliations:** 1 Department of Neurosurgery, HwaMei Hospital, University of Chinese Academy of Sciences, Ningbo, China; 2 Department of Emergency, Ningbo Yinzhou No. 2 Hospital, Ningbo, China; 3 Operating Room, Ningbo Yinzhou No. 2 Hospital, Ningbo, China; 4 Department of Experimental Medical Science, HwaMei Hospital, University of Chinese Academy of Sciences, Ningbo, China

**Keywords:** parkinson’s disease, microglia, inflammasome, edaravone, polarization

There was a mistake in Figure 6A as published. The resolution of the immunofluorescence image was insufficient and did not meet the required quality for clear presentation. The corrected Figure 6 appears below.

**FIGURE 6:**
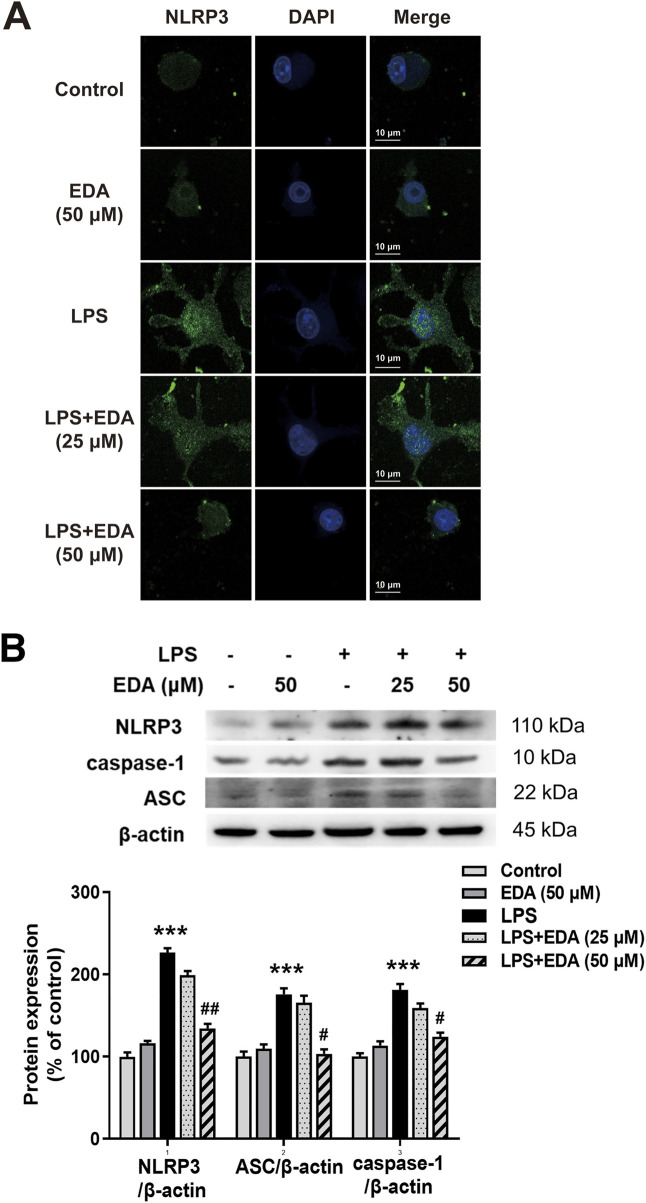
EDA Inhibited NLRP3 inflammasome activation *in vitro*. BV-2 cells were treated with EDA for 1 h followed by the application of LPS (100 ng/mL) for 24 h. **(A)** Immunofluorescence detection of NLRP3 inflammasome in BV-2 cells were performed. Scale bar = 10 μm. **(B)** The protein expressions of NLRP3, ASC and caspase-1 were detected by Western blot assay. The ratio of densitometry values of NLRP3, ASC and caspase-1 with β-actin were assessed and normalized to each respective control cultures. Data were the mean ± SEM from six independent experiments performed in triplicate. ***p < 0.001 compared with control cultures; #p < 0.05, #p < 0.01 compared with LPS-treated cultures.

The original article has been updated.

